# Detecting a Local Cohort Effect for Cancer Mortality Data Using a Varying Coefficient Model

**DOI:** 10.2188/jea.JE20140218

**Published:** 2015-11-05

**Authors:** Tetsuji Tonda, Kenichi Satoh, Ken-ichi Kamo

**Affiliations:** 1 Faculty of Management and Information Systems, Prefectural University of Hiroshima, Hiroshima, Japan; 1 県立広島大学 経営情報学部; 2 Research Institute for Radiation Biology and Medicine, Hiroshima University, Hiroshima, Japan; 2 広島大学 原爆放射線医科学研究所; 3 Center for Medical Education, Sapporo Medical University, Sapporo, Japan; 3 札幌医科大学 医療人育成センター

**Keywords:** birth cohort effect, cancer mortality in Japan, varying coefficient model

## Abstract

**Background:**

Cancer mortality is increasing with the aging of the population in Japan. Cancer information obtained through feasible methods is therefore becoming the basis for planning effective cancer control programs. There are three time-related factors affecting cancer mortality, of which the cohort effect is one. Past descriptive epidemiologic studies suggest that the cohort effect is not negligible in cancer mortality.

**Methods:**

In this paper, we develop a statistical method for automatically detecting a cohort effect and assessing its statistical significance for cancer mortality data using a varying coefficient model.

**Results:**

The proposed method was applied to liver and lung cancer mortality data on Japanese men for illustration. Our method detected significant positive or negative cohort effects. The relative risk was 1.54 for liver cancer mortality in the cohort born around 1934 and 0.83 for lung cancer in the cohort born around 1939.

**Conclusions:**

Cohort effects detected using the proposed method agree well with previous descriptive epidemiologic findings. In addition, the proposed method is expected to be sensitive enough to detect smaller, previously undetected birth cohort effects.

## Abstract (ja)

**【背景】:**

がん死亡リスクは加齢とともに増大傾向にあり、高齢化が進む日本において、がん死亡リスクの評価や予測は重要な課題である。がん死亡リスクに影響を及ぼす時間要因は3つあり、その1つが出生コホート効果である。これまで、記述疫学研究により日本のがん死亡リスクにおけるコホート効果の存在が報告されている。

**【方法】:**

本研究では、出生コホート効果を自動検出し、その効果を統計的に評価するための方法を開発する。

**【結果】:**

提案法を日本人男性の肝臓がんと肺がんに関する死亡データに適用した。その結果、

肝臓がんについては1934年前後の出生コホートに有意な正の効果が認められ、その相対危険度は最大で約1.54倍であった。肺がんについては、1939年前後の出生コホートに有意な負の効果が認められ、その相対危険度は最小で約0.83倍であった。

**【結論】:**

提案法で検出された出生コホート効果は、記述疫学研究の結果とほぼ一致しており、提案法の有用性が確認された。提案法の適用により、これまで見落とされている可能性がある弱い出生コホート効果の検出が期待される。

## INTRODUCTION

In Japan, cancer has been the leading cause of death since 1981, becoming a serious concern in the aging society. Precise trends in cancer risk must be identified to develop efficient cancer control programs.

**Figure 1.**
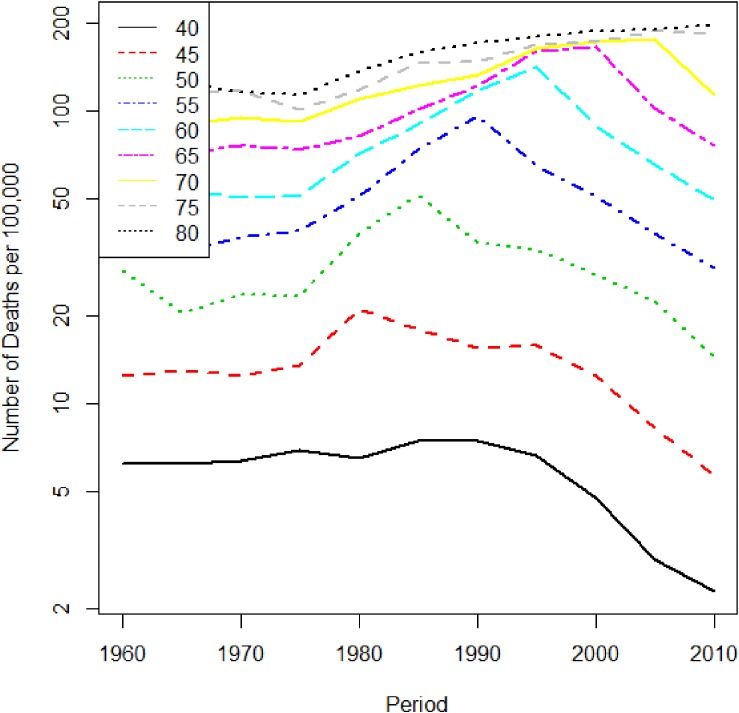
Liver cancer mortality trend in Japanese males by age. The horizontal axis is period, and the vertical axis is the mortality rate per 100 000 person years.

Three time-dependent factors affect cancer mortality: age, period, and birth cohort. We illustrate these effects with liver cancer mortality data in Japanese men as a typical example. Cancer mortality data can be obtained from the website of the National Cancer Center in Japan.^1^ The data are tabulated by 5-year age categories. Figure 1 shows time trends of mortality by age, which allows us to understand the age and period effects. Liver cancer mortality increases with age but has decreased in the recent period.

**Figure 2.**
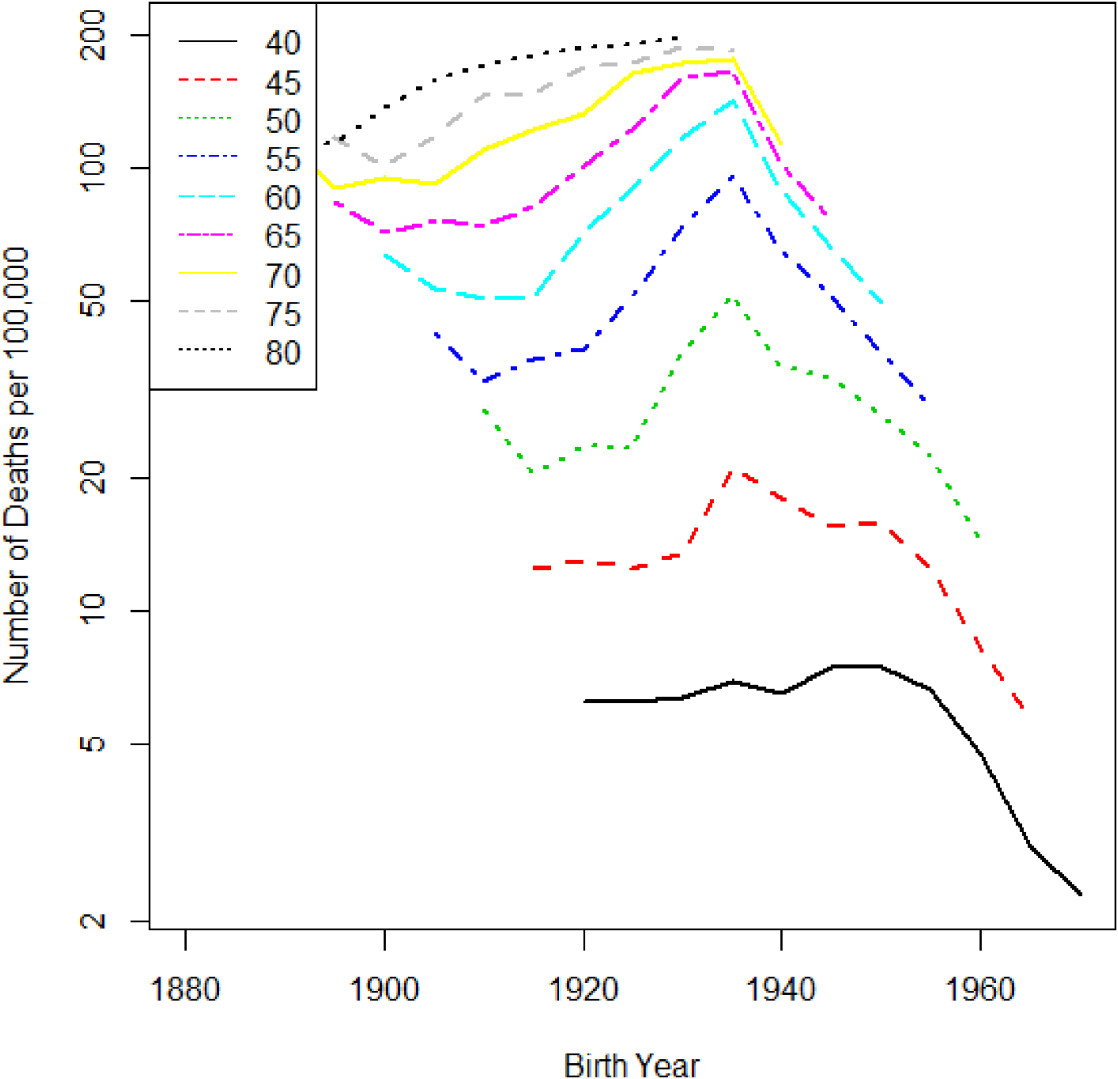
Liver cancer mortality trend in Japanese males by age. The horizontal axis is birth year, and the vertical axis is the mortality rate per 100 000 person years.

There are also characteristic local changes for certain periods by age. Figure 2 shows the trend of liver cancer, but with birth year rather than period; local changes occur among subjects whose birth year is around 1935. This is regarded as a birth cohort effect. In many past studies, it has been pointed out that the cohort born in the 1930s has a high risk of liver cancer.^2^^,^^3^ The reason for this is thought to be the high prevalence of hepatitis C virus infection in Japan.^4^^,^^5^ The cohort effect for liver cancer mortality in Japanese males is easy to be identified, but cohort effects are typically not discernible to the eye.

There are statistical methods for assessing age, period, and cohort effects simultaneously, such as age-period-cohort (APC) analysis.^6^ APC analysis suffers from a model identification problem due to the exact linear dependency among the three variables: cohort = period − age. As a result, it is not generally possible to estimate the three effects separately without additional constraints to identify the model.^7^^,^^8^ To overcome this problem, several approaches to estimating the three factors have been proposed under various assumptions or constraints. However, differences in assumptions or constraints often produce inconsistent results.^9^ Keyes et al^10^ compared three approaches: the traditional constraint-based regression technique,^6^ the Holford model,^11^^,^^12^ and the median polish technique,^13^ using data on the obesity prevalence in the United States from 1971–2006.^14^^–^^16^ The results of the different approaches regarding cohort effect were not consistent. They considered that this inconsistency was due to differences in conceptual definitions of cohort effect as estimated by the three approaches.

As another approach, Kamo, Satoh, and Tonda^17^ proposed visualizing mortality risk using mortality data tabulated by age and period. They visualized the cancer mortality risk by the surface of the age-period plane and suggested searching for a birth cohort effect based on the surface. The main objective of their method is to elucidate the characteristics of cancer trends using visualization, not to statistically judge whether cohort effects exist or not. While their method is useful for discerning a global trend in age and/or period, it might be difficult to identify a birth cohort effect empirically, except in extreme cases, such as the birth cohort effect with liver cancer mortality in Japanese males.

We developed a statistical method to detect a cohort effect automatically and assess its statistical significance for cancer mortality data using a varying coefficient model. In general, there are two types of cohort effects: global and local. In this paper, we focus on detecting a local change as a cohort effect. It is especially important to describe the birth cohort effect in a statistical model for prediction of future cancer mortality. Automatic detection is important, because evaluation by eyesight carries the risk of errors due to the researcher’s subjective expectations or bias.

In the present paper, we introduce a varying coefficient model and construct a method for estimating the varying coefficient with its statistical evaluation. We then apply the proposed method to data on liver and lung cancer mortality in Japanese males. Finally, we review possible reasons for the birth cohort effect detected by the proposed method and evaluate the method’s performance with its instructions for use in practice.

## METHODS

### Varying coefficient model

**Figure 3.**
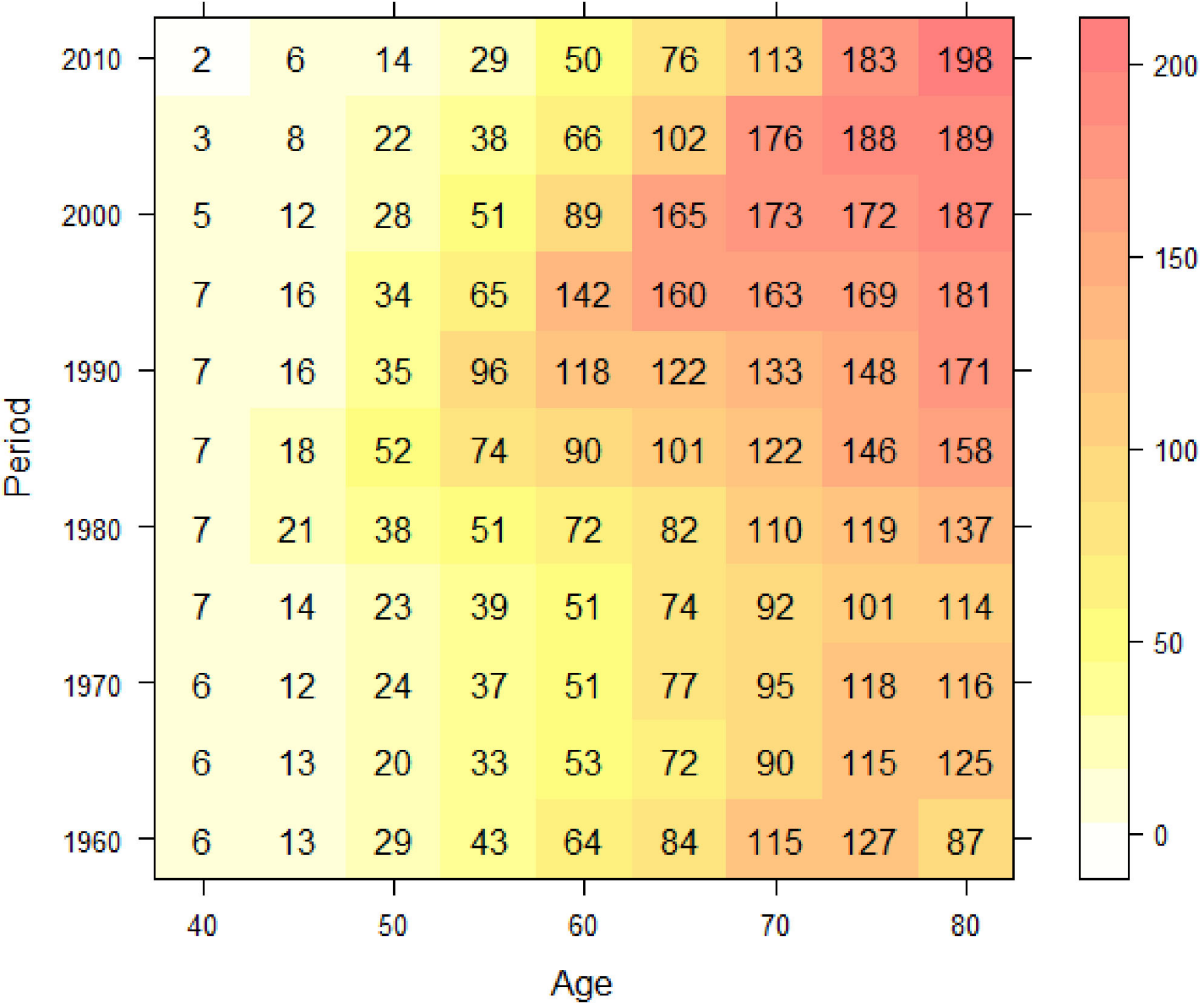
Tabulated mortality rate per 100 000 subjects by age and period for liver cancer mortality of Japanese males.

Let (*z**_a_*_,_*_p_*, *d_a_*_,_*_p_*) denote the set of population person-time and observed number of deaths for age *a* during period *p*. The observed number of deaths is assumed to follow a Poisson distribution,

$$d_{a,p}∼\text{Poisson}(z_{a,p}\lambda_{a,p}),\;\log\lambda_{a,p}=\beta_{0}(a,p),$$

where *β*_0_(*a*, *p*) is the regression coefficient varying with age *a* and period *p*. Regression coefficients that vary with time, geographical location, or other important covariates are generally called varying coefficients, and the varying coefficient model was proposed by Hastie and Tibshirani.^18^ The varying coefficient transformed to the risk scale, exp(*β*_0_(*a*, *p*)), represents a surface of mortality risk on the age-period plane. Note that the mortality rate by age and period is regarded as a crude estimate of exp(*β*_0_(*a*, *p*)). Figure 3 shows the mortality rate per 100 000 person-years by 5-year age group and 5-year period with gradations represented by a heat map. Note that a cohort with birth year *c* lies on the diagonal line *p* − *a* = *c* in Figure 3. If a birth cohort effect exists, it appears as a higher or lower diagonal line. However, it is not easy to identify such a trend using the mesh-type mapping in Figure 3. We estimate the varying coefficient to present it as a smooth mapping. There are several ways to estimate varying coefficients. One is nonparametric method such as kernel smoothing^19^^,^^20^ and geographical weighted regression (GWR).^21^ Another is parametric method based on an interaction model.^22^

The GWR model^21^ is widely used for spatial data with a continuous outcome. Nakaya et al^23^ extended the GWR model to a geographically weighted Poisson regression (GWPR) model for spatial count data. The GWPR is implemented in the GWR4.0 software^24^ and the spgwr package in R.^25^ Regarding the set of age and period as a virtual geographical location, Kamo et al^17^ applied the GWPR to visualize cancer mortality risks as a surface on the age-period plane. Nonparametric estimation based on the GWPR is useful to grasp the age-period trend of mortality, but it is often difficult to detect a birth cohort effect visually based solely on the contours. We therefore constructed a parametric model to identify the birth cohort effect automatically using statistical evaluation.

### Estimation of varying coefficient

Satoh and Yanagihara^22^ proposed a parametric method for estimating varying coefficients for longitudinal data with continuous outcomes. Their method was extended to longitudinal data with a discrete outcome^26^ as well as to spatial data.^27^^–^^29^ For cancer mortality data, Kamo, Satoh, and Tonda^17^ modeled *β*_0_(*a*, *p*) by interactions of polynomials in age and period; that is, *β*_0_(*a*, *p*) = $\theta^{\prime }$ ***x***(*a*, *p*), where θ is an *m*-dimensional vector of unknown parameters and ***x***(*a*, *p*) is an *m*-dimensional basis vector whose elements are interactions of polynomials in age and period. For example, interactions of cubic polynomials produce the basis

$$\begin{array}{rl} x(a,p) & =(1,a,a^{2},a^{3},p,ap,a^{2}p,a^{3}p,p^{2},ap^{2}a^{2}p^{2},\\ & \;a^{3}p^{2},p^{3},ap^{3}a^{2}p^{3},a^{3}p^{3}{)}^{\prime }. \end{array}$$

The interaction terms in ***x***(*a*, *p*) describes not only age and period effects but also a global trend on cohort effect. To model a local change of cohort effect, we here add a basis of normal density with mean *μ_c_* and variance $\sigma_{c}^{2}$ into *β*_0_(*a*, *p*); that is,

$$\begin{array}{rl} & \beta_{0}(a,p)=\theta^{\prime }x(a,p)+\beta_{c}\phi (\mu_{c},\sigma_{c}^{2}),\\ & \phi (\mu_{c},\sigma_{c}^{2})=\frac{1}{\sqrt{2\pi \sigma_{c}^{2}}}\exp\left(-\frac{(p-a-\mu_{c}{)}^{2}}{2\sigma_{c}^{2}}\right). \end{array}$$

Note that *μ_c_* and *σ_c_* denote the center and range of birth cohort. In this paper, we regard the cohort effect as the local change if the value of *σ_c_* is within a period of about 5% for a whole range of birth year. We then consider the relative risk $\exp(\beta_{c}\phi (\mu_{c},\sigma_{c}^{2}))$ as the cohort effect. A statistical test for a cohort effect is also available based on a test for the usual null hypothesis on *β_c_*.

The number of deaths is observed at *n* combinations of age and period. Then, let $\left\{(a_{j},p_{j}),j=1,\ldots ,n\right\}$ be a set of observed age and period, where *a_j_* and *p_j_* denote the *j*th combination of age and period. For a fixed *μ_c_* and *σ_c_*, the unknown parameters θ and *β_c_* are estimated by maximizing the log-likelihood:

$$\begin{array}{rl} & \left(\begin{array}{c} \hat{\theta \;}\;\\ {\hat{\beta }}_{c} \end{array}\right)=\arg\max\ell (\theta ,\beta_{c}|\mu_{c},\sigma_{c}^{2}),\\ & \ell (\theta ,\beta_{c}|\mu_{c},\sigma_{c}^{2})=\sum_{j=1}^{n}\log f(d_{a_{j},p_{j}}|z_{a_{j},p_{j}}), \end{array}$$

where $\log f(d|z)=d(\log z+\beta_{0}(a,p))-ze^{\beta_{0}(a,p)}-\log d!$. In addition, ${\hat{\beta }}_{0}(a,p)={\hat{\theta \;}\;}^{\prime }x(a,p)+{\hat{\beta }}_{c}\phi (\mu_{c},\sigma_{c}^{2})$ is obtained. Using $-2\ell (\hat{\theta \;}\;,{\hat{\beta }}_{c}|\mu_{c},\sigma_{c}^{2})$ as an index of goodness-of-fit, *μ_c_* and *σ_c_* are determined by minimizing $-2\ell (\hat{\theta \;}\;,{\hat{\beta }}_{c}|\mu_{c},\sigma_{c}^{2})$.

## RESULTS

We evaluated the performance of our proposed method for detecting a cohort effect using liver and lung cancer mortality in Japanese males. We choose these two cancers as examples because past epidemiological studies already reported the possibility of birth cohort effects.

### Liver cancer mortality

**Figure 4.**
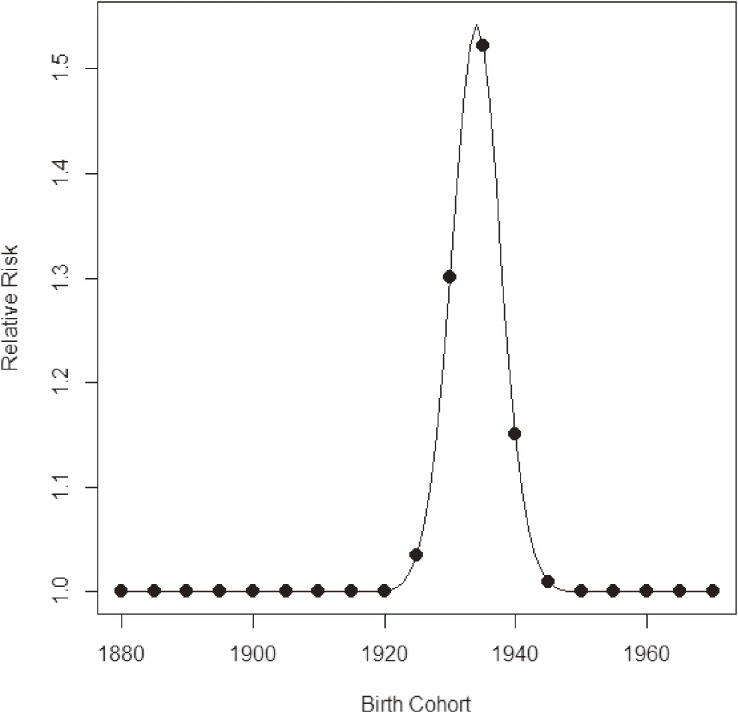
Estimated relative risks by birth cohort for liver cancer in Japanese males.

**Figure 5.**
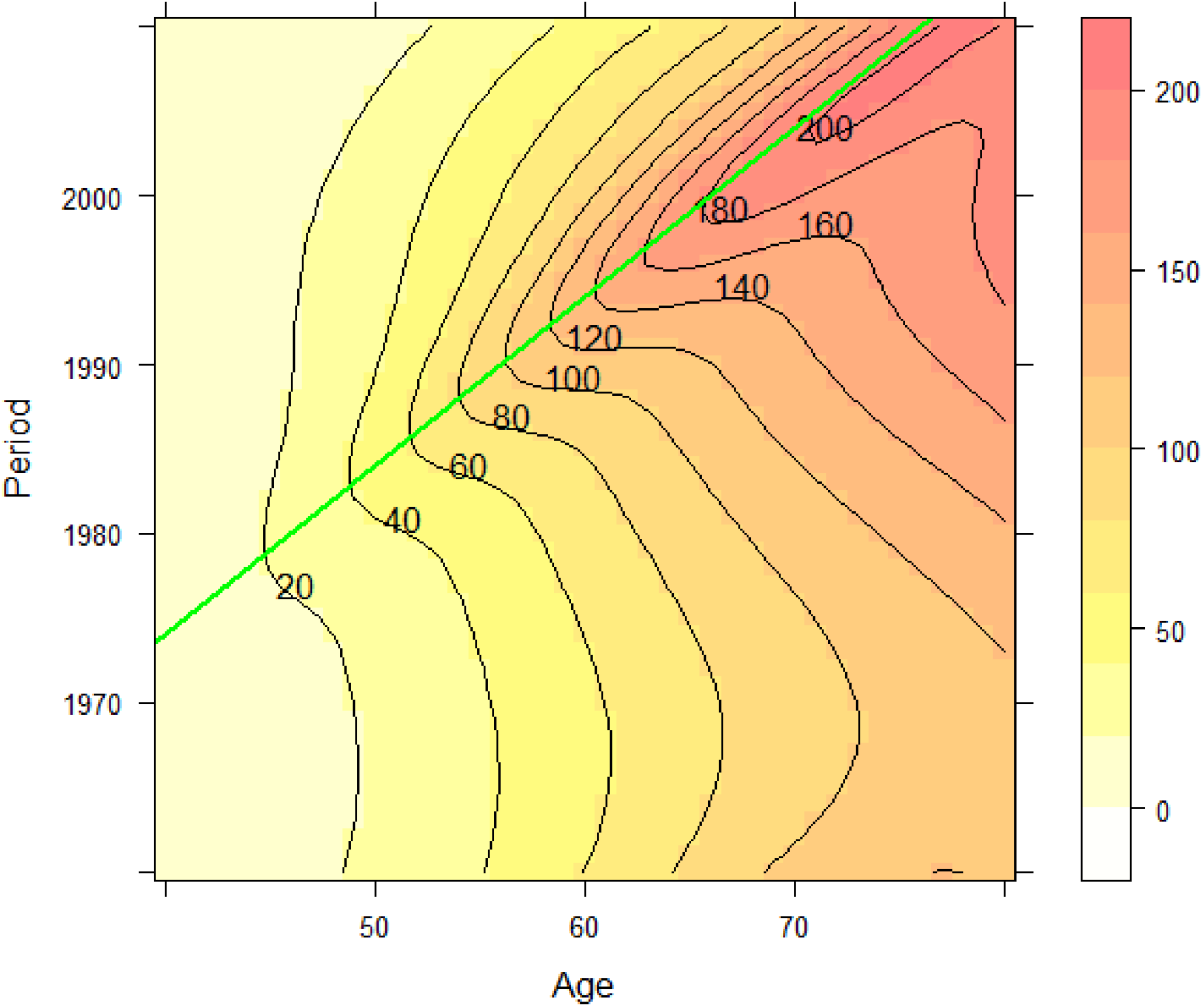
Estimated surface of liver cancer mortality in Japanese males using the proposed method. The horizontal and vertical axes denote age and period, respectively. Contour lines denote mortality rate per 100 000 persons in each year. The diagonal line denotes the 1934 birth cohort, which was detected as the center of cohort.

As mentioned previously, many past studies have noted that the cohort born around 1935 has a high risk of liver cancer. We used our method to search for a cohort effect automatically. We estimated *β*_0_(*a*, *p*) using ***x***(*a*, *p*) based on the interactions of cubic polynomials basis. Minimizing the values of $-2\ell (\hat{\theta },{\hat{\beta }}_{c}|\mu_{c},\sigma_{c}^{2})$ for several sets of *μ_c_* and *σ_c_*, the best-fitted model was (*μ_c_*, *σ_c_*) = (1934, 4). Table 1 gives the estimated parameters corresponding to θ for the interactions of cubic polynomials basis and *β_c_* for (*μ_c_*, *σ_c_*) = (1934, 4). Because the estimate of *β_c_* was about 4.35 and the statistical test of the null hypothesis that *β_c_* = 0 was rejected with a high level of significance, we were able to declare a significant positive effect (increased risk) for the birth cohort around 1934. Figure 4 shows the relative risks corresponding to this birth cohort effect. The maximum relative risk was about 1.54. Figure 5 shows the estimated cancer mortality surface describing a smooth mapping against Figure 3. The diagonal line denotes the 1934 birth cohort, which was detected as the center of the cohort. The positive birth cohort effect can be seen around the diagonal line for the 1934 birth cohort.

**Table 1.** The estimated parameters corresponding to ***θ*** and *β_c_* in the case (*μ_c_*, *σ_c_*) = (1934, 4) for liver cancer mortality in Japanese males

Parameter		Estimate	Std. Error	z-value	*P*-value
***θ***^a^	(Intercept)	−7.1481	0.0072	−989.323	<0.001

	*a*	0.6275	0.0094	66.431	<0.001

	*a*^2^	−0.2347	0.0040	−58.867	<0.001

	*a*^3^	0.0446	0.0034	13.082	<0.001

	*p*	0.2245	0.0076	29.467	<0.001

	*p*^2^	−0.0632	0.0021	−29.515	<0.001

	*p*^3^	−0.0443	0.0016	−27.745	<0.001

	*ap*	0.0174	0.0100	1.744	0.081

	*ap*^2^	0.0336	0.0034	10.031	<0.001

	*ap*^3^	0.0021	0.0021	0.998	0.318

	*a*^2^*p*	−0.0101	0.0050	−2.037	0.042

	*a*^2^*p*^2^	0.0013	0.0015	0.892	0.373

	*a*^2^*p*^3^	−0.0010	0.0011	−0.868	0.385

	*a*^3^*p*	0.0001	0.0036	0.029	0.977

	*a*^3^*p*^2^	−0.0039	0.0012	−3.227	0.001

	*a*^3^*p*^3^	0.0021	0.0008	2.722	0.007

*β_c_*		4.3470	0.1148	37.872	<0.001

^a^The elements of ***θ*** are coefficients of interactions in the cubic polynomial basis.

### Lung cancer mortality

**Figure 6.**
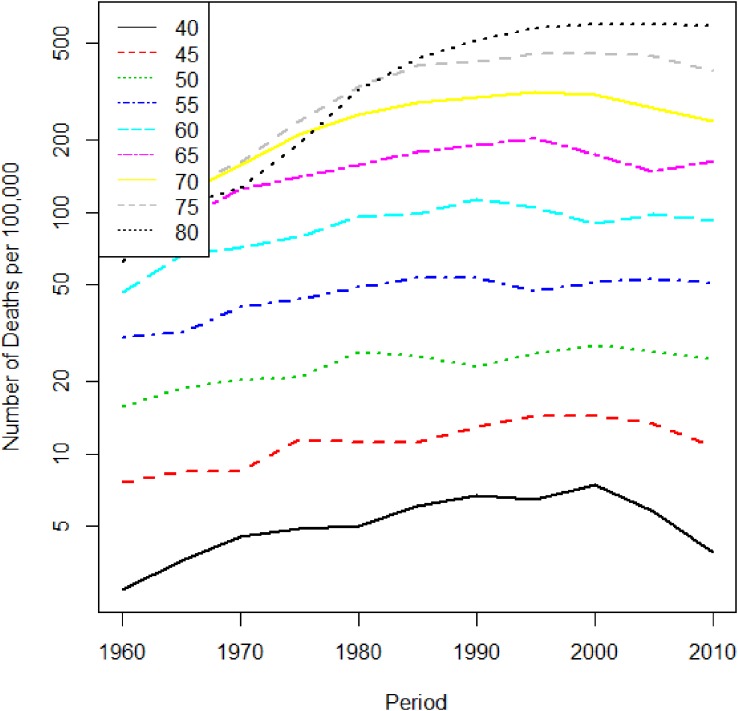
Lung cancer mortality trend in Japanese males by age. The horizontal axis is period, and the vertical axis is the mortality rate per 100 000 person years.

**Figure 7.**
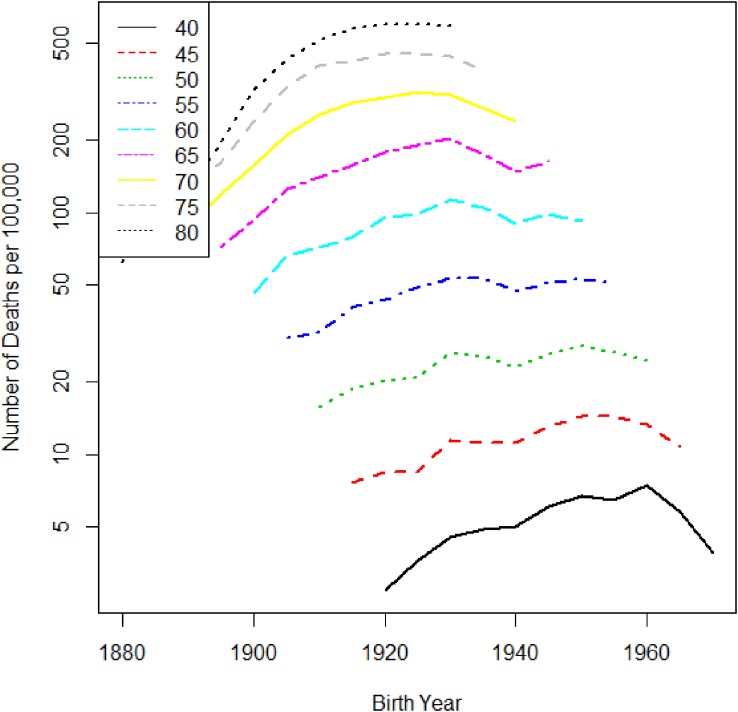
Lung cancer mortality trend for Japanese males by age. The horizontal axis is birth year, and the vertical axis is the mortality rate per 100 000 person years.

**Figure 8.**
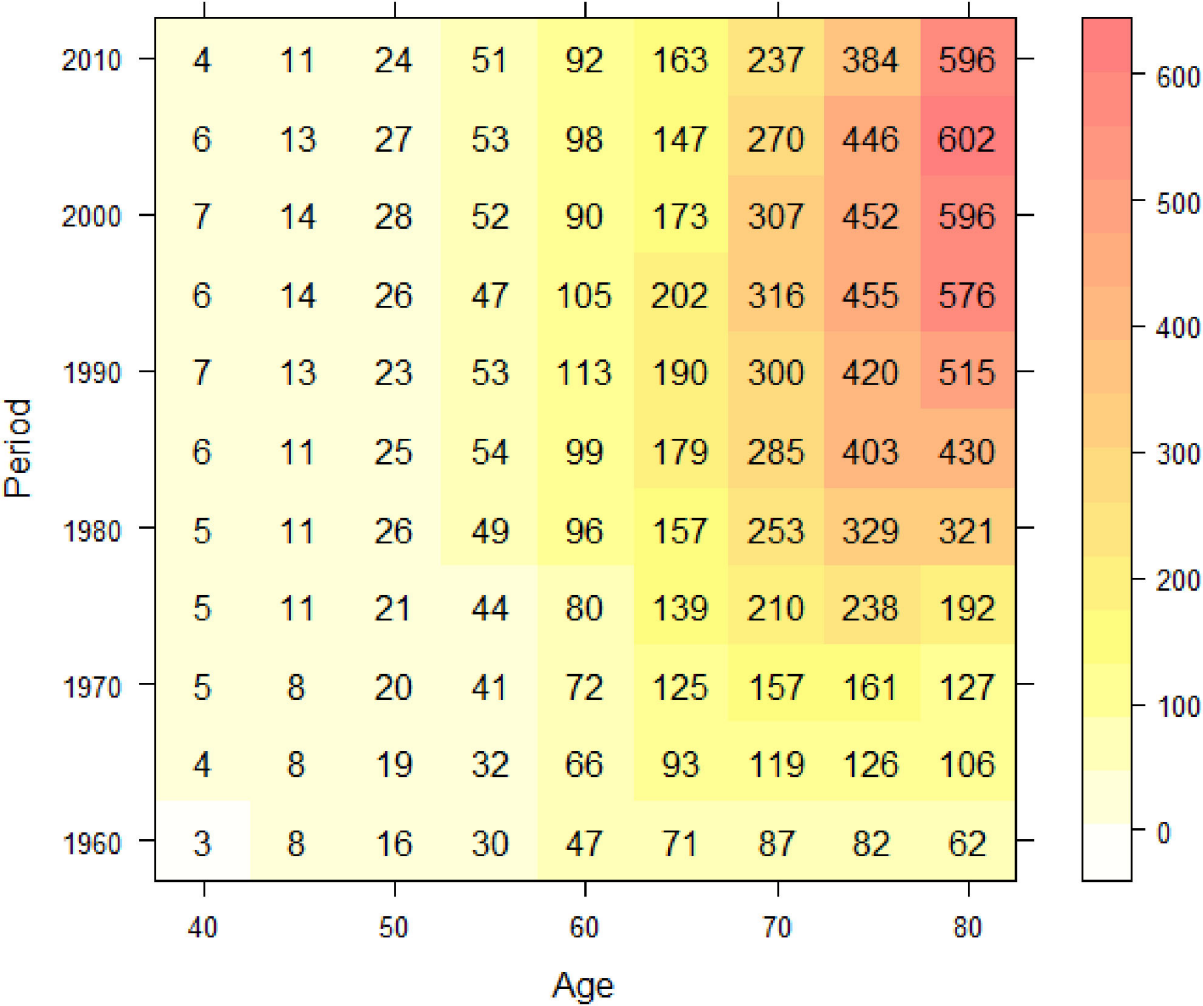
Tabulated mortality rate per 100 000 subjects by age and period for lung cancer mortality in Japanese males.

**Figure 9.**
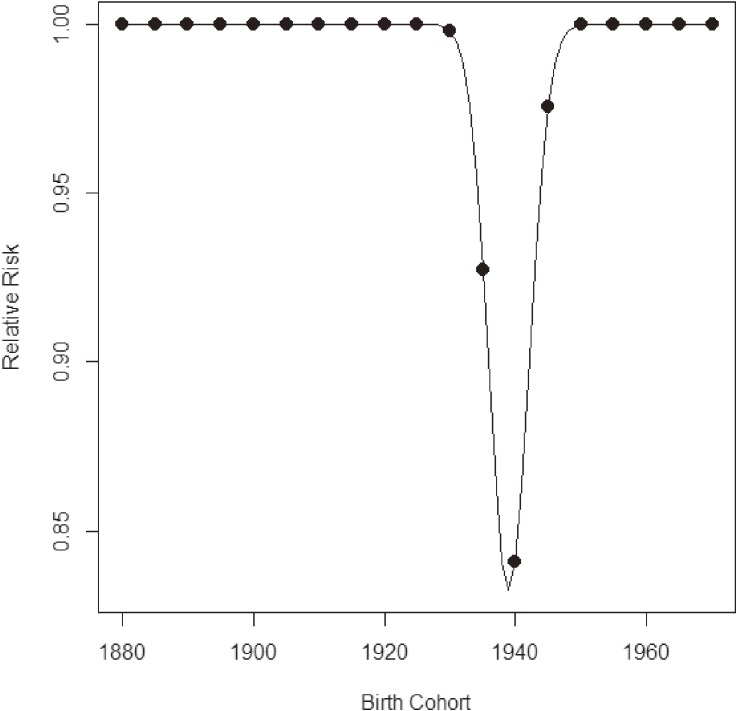
Estimated relative risks by birth cohort for lung cancer mortality in Japanese males.

**Figure 10.**
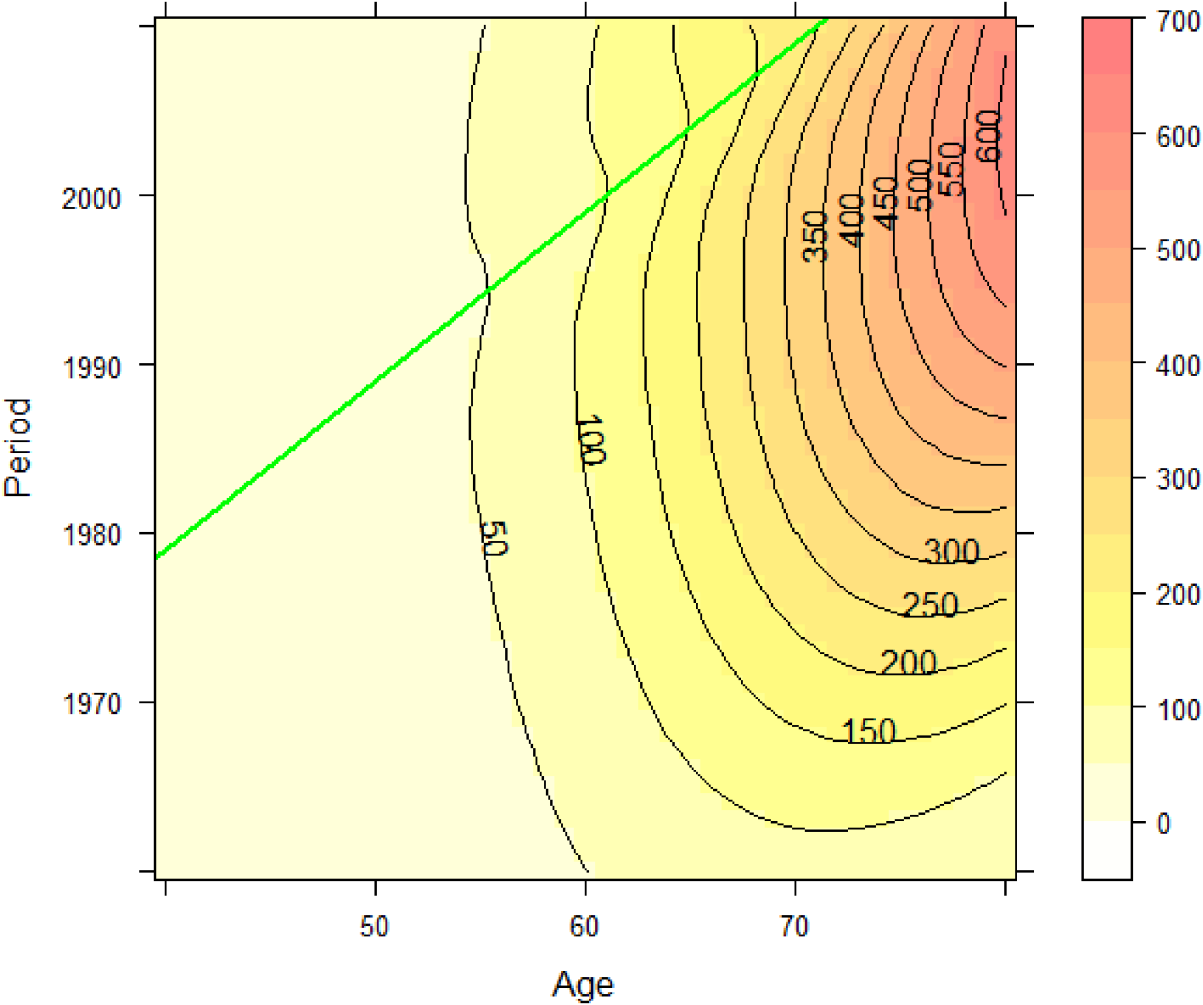
Estimated surface of lung cancer mortality in Japanese males by the proposed method. The horizontal and vertical axes denote age and period, respectively. Contour lines denote mortality rate per 100 000 persons in each year. The diagonal line denotes the 1939 birth cohort, which was detected as the center of cohort.

A previous descriptive epidemiologic study^30^^,^^31^ noted the possibility of a small birth cohort effect with a local peak around the late 1920s and a declining trend until the late 1930s. Figure 6 and Figure 7 show time trends of lung cancer mortality in Japanese males by age. The horizontal axes in Figure 6 and Figure 7 are calendar year and birth year, respectively. From these figures, it is difficult to identify a cohort effect intuitively unless one is a highly experienced epidemiologist. Therefore, we applied our method to search for a cohort effect automatically. Figure 8 shows the data tabulated by age and period colored with the corresponding heat map for lung cancer mortality in Japanese males. We estimated *β*_0_(*a*, *p*) using ***x***(*a*, *p*) based on the interactions of cubic polynomials basis. Minimizing the values of $-2\ell (\hat{\theta },{\hat{\beta }}_{c}|\mu_{c},\sigma_{c}^{2})$ for several sets of *μ_c_* and *σ_c_*, the best-fitted model was (*μ_c_*, *σ_c_*) = (1939, 3). Table 2 gives the estimated parameters corresponding to θ for the interactions of cubic polynomials basis and *β_c_* for (*μ_c_*, *σ_c_*) = (1939, 3). Because the estimate of *β_c_* was about −1.38 and a statistical test of the hypothesis *β_c_* = 0 was highly significant, we were able to declare a significant negative effect (decreased risk) around the 1939 birth cohort. Figure 9 shows the relative risks in the detected birth cohort effect. The minimum relative risk for the birth cohort was about 0.83. Figure 10 shows the estimated cancer mortality surface describing a smooth mapping against Figure 8. The diagonal line denotes the 1939 birth cohort, which was detected as the center of the cohort. The negative birth cohort effect can be seen around the diagonal line for the 1939 birth cohort.

**Table 2.** The estimated parameters corresponding to ***θ*** and *β_c_* in the case (*μ_c_*, *σ_c_*) = (1939, 3) for lung cancer mortality in Japanese males

Parameter		Estimate	Std. Error	z-value	*P*-value
***θ***^a^	(Intercept)	−6.8839	0.0057	−1200.643	<0.001

	*a*	1.2338	0.0081	151.528	<0.001

	*a*^2^	−0.1676	0.0038	−44.632	<0.001

	*a*^3^	−0.0491	0.0029	−16.700	<0.001

	*p*	0.1238	0.0067	18.364	<0.001

	*p*^2^	−0.0649	0.0020	−32.567	<0.001

	*p*^3^	0.0003	0.0014	0.218	0.827

	*ap*	0.0187	0.0086	2.179	0.029

	*ap*^2^	−0.0291	0.0031	−9.295	<0.001

	*ap*^3^	0.0079	0.0020	4.002	<0.001

	*a*^2^*p*	0.0436	0.0045	9.672	<0.001

	*a*^2^*p*^2^	−0.0123	0.0015	−8.438	<0.001

	*a*^2^*p*^3^	−0.0007	0.0010	−0.724	0.469

	*a*^3^*p*	0.0134	0.0032	4.127	<0.001

	*a*^3^*p*^2^	0.0039	0.0012	3.271	0.001

	*a*^3^*p*^3^	−0.0007	0.0008	−0.875	0.382

*β_c_*		−1.3787	0.0835	−16.517	<0.001

^a^The elements of ***θ*** are coefficients of interactions in the cubic polynomial basis.

## DISCUSSION

In this paper, we deal with the data for cancer mortality. Analysis using data for cancer mortality is suitable for the first proposal of a new statistical procedure because the quality of the data is quite high in Japan. If established epidemiological knowledge for the cohort effect can be detected using this method, then this method can be considered to work well. Moreover, this method may useful in evaluating incidence of cancer or other diseases. From the viewpoint of cancer control programs, information on not only cancer mortality but also cancer incidence is particularly important. However, since cancer incidence data tend to have issues with completeness and quality, we chose a more robust dataset for our evaluation.^32^ Similarly, when we apply this method to other diseases, we have to pay attention to the properties of the data.

The proposed method detected a significant positive effect for the cohort born around 1934 for liver cancer mortality in Japanese males. This result agrees well with previous epidemiologic studies.^2^^,^^3^^,^^33^ Yoshimi and Sobue^2^ and Imamura and Sobue^3^ reported that liver cancer mortality exhibits the most notable birth cohort effect among cancer sites in Japan. As mentioned previously, this positive cohort effect is attributed to the markedly high prevalence of hepatitis C virus infection in the cohort born around 1935.^4^^,^^5^

For lung cancer mortality in Japanese males, the proposed method detected a significant negative effect for the cohort born around 1939. A possible reason for such an effect was discussed by Marugame et al.^34^ It is known that cigarette smoking relates to lung cancer. From the end of World War II to the beginning of the Japanese post-World War II economic expansion, Japan experienced an extreme shortage of cigarettes. Therefore, men born during the 1930s had less opportunity to begin smoking during adolescence, so their cohort showed a corresponding dip in smoking prevalence. It is difficult to detect this birth cohort effect directly from the figures without careful observation, demonstrating the utility of the proposed method. Takahashi et al^35^ analyzed mortality data on lung cancer in Japan using an APC model based on that of Holford.^11^^,^^36^ Based on a figure showing changing patterns of non-linear birth cohort effect, they also suggested the existence of a local change at the end of the 1930s. However, their suggestion is based on visual consideration of the figure without rigorous statistical evaluation.

We discuss the instructions for use of the proposed method in practice using cancer mortality data on other sites. Applying the proposed method to stomach cancer mortality in Japanese males, the optimal center and range of cohort were (*μ_c_*, *σ_c_*) = (1920, 21). This range of cohort seems to be too wide to be considered a local change. We therefore consider that no local birth cohort effect exists. As mentioned previously, there are two types of cohort effects: global trends, which denote a gradual change over time, and local changes within a short time. In the proposed method, the interaction terms of ***x***(*a*, *p*) is regarded as modeling a global trend on cohort effect. The normal density basis, $\phi (\mu_{c},\sigma_{c}^{2})$, models a local change on cohort effect, if such a change exists. However, if no local change exists, $\phi (\mu_{c},\sigma_{c}^{2})$ works for fitting a global trend on cohort effect together with the interaction terms of ***x***(*a*, *p*), and the range of the cohort, $\sigma_{c}^{2}$, tends to be large. In this case, it is natural to consider that no local change exists. For cancer mortality at other sites, except for the rectum, the results were similar to that for stomach cancer in that the optimal range of the cohort tended to be wide. These results show no cohort effect for cancer mortality at most sites. The findings for rectal cancer showed that the optimal center and range of cohort was (*μ_c_*, *σ_c_*) = (1921, 7). The range of the cohort seems to be not so wide, suggesting the potential existence of a cohort effect. Note that the estimate of *β_c_* was about −4.24, and the minimum relative risk with birth cohort was about 0.79.

In many past epidemiologic studies, cohort effects have been inferred by evaluating longitudinal behavior. However, such a classical method is subject to error due to the researcher’s bias or subjectivity influencing the results. One solution to this problem is to judge the effect automatically using a statistical method, which allows for objective detection of a cohort effect. In our analyses of liver and lung cancer in Japanese males, we successfully identified birth cohort effects automatically, and our results agree with those of past epidemiologic findings. It is expected that this method will prove useful in identifying small or previously undetected cohort effects. The method should also prove useful for developing a statistical model to predict future cancer mortality.

In this paper we focused on detecting a single birth cohort effect that changes locally. In previous descriptive studies, some epidemiologists noted the possibility of multiple birth cohort effects; for example, lung cancer in males shows another cohort effect in the late 1920s, which can be seen in Figure 10 as a second candidate cohort effect. Our method should be revised to account for multiple cohort effects in the future.

## ONLINE ONLY MATERIAL

Abstract in Japanese.

## References

[1] Ministry of Health, Labour and Welfare. Vital Statistics Japan, 1958–2012. Center for Cancer Control and Information Services, National Cancer Center, Japan. Available from: http://www.ncc.go.jp/.

[2] Yoshimi I, Sobue T. Mortality trend of liver cancer in Japan 1960–2000. Jpn J Clin Oncol. 2003;33:202–3. 12816081

[3] Imamura Y, Sobue T. Mortality trend of colon, rectal, liver, “gallbladder and biliary tract” and pancreas cancer in Japan by birth cohort. Jpn J Clin Oncol. 2004;34:491–3. 10.1093/jjco/hyh085 15371470

[4] Ishiguro S, Inoue M, Tanaka Y, Mizokami M, Iwasaki M, Tsugane S; JPHC Study Group. Impact of viral load of hepatitis C on the incidence of hepatocellular carcinoma: a population-based cohort study (JPHC Study). Cancer Lett. 2011;300:173–9. 10.1016/j.canlet.2010.10.002 21035947

[5] Tanaka H, Uera F, Tsukuma H, Ioka A, Oshima A. Distinctive change in male liver cancer incidence rate between the 1970s and 1990s in Japan: comparison with Japanese-Americans and US whites. Jpn J Clin Oncol. 2007;37:193–6. 10.1093/jjco/hyl148 17332055

[6] Mason KO, Mason WM, Winsborough HH, Poole WK Some methodological issues in cohort analysis of archival data. Am Sociol Rev. 1973;38:242–58. 10.2307/2094398

[7] Kupper LL, Janis JM, Karmous A, Greenberg BG. Statistical age-period-cohort analysis: a review and critique. J Chronic Dis. 1985;38:811–30. 10.1016/0021-9681(85)90105-5 4044767

[8] Mason WM, Wolfinger NH. Cohort Analysis. In: Smelser NJ, Baltes PB, editors. International Encyclopedia of the Social and Behavioral Sciences. Amsterdam: Elsevier Science; 2001. p. 2189–94.

[9] Frenk SM, Yang YC, Land KC. Assessing the Significance of Cohort and Period Effects in Hierarchical Age-Period-Cohort Models: Applications to Verbal Test Scores and Voter Turnout in U.S. Presidential Elections. Soc Forces. 2013;92(1):221–48. 10.1093/sf/sot066 25392566 PMC4226416

[10] Keyes KM, Utz RL, Robinson W, Li G. What is a cohort effect? Comparison of three statistical methods for modeling cohort effects in obesity prevalence in the United States. 1971–2006. Soc Sci Med. 2010;70:1100–8. 10.1016/j.socscimed.2009.12.018 20122771 PMC3469580

[11] Holford TR. The estimation of age, period and cohort effects for vital rates. Biometrics. 1983;39:311–24. 10.2307/2531004 6626659

[12] Holford TR. Analysing the temporal effects of age, period and cohort. Stat Methods Med Res. 1992;1:317–37. 10.1177/096228029200100306 1341663

[13] Keyes KM, Li G A comprehensive approach to age-period-cohort analysis [abstract]. Am J Epidemiol. 2008;167:S109.

[14] National Center for Health Statistics Plan and Operation of the Health and Nutritional Examination Survey, United States: 1971–1973. Programs and Collection Procedure. Vital and Health Statistics. 1978;10:1–46. 4347506

[15] National Center for Health Statistics. Plan and Operation of the Third National Health and Nutritional Examination Survey, 1988–1994. Series 1: programs and collection procedures. Vital Health Stat 1. 1994;(32):1–407. 7975354

[16] National Center for Health Statistics. Analytic and Reporting Guidelines: The National Health and Nutrition Examination Survey (NHANES). 2005. Available from: http://www.cdc.gov/nchs/data/nhanes/nhanes_03_04/nhanes_analytic_guidelines_dec_2005.pdf.

[17] Kamo K, Satoh K, Tonda T Cancer mortality risk visualization on age-period plane. Proc Inst Stat Math. 2011;59:217–37 (in Japanese).

[18] Hastie T, Tibshirani R Varying-coefficient models. J R Stat Soc, B. 1993;55:757–96.

[19] Wand MP, Jones MC. Kernel Smoothing. New York: Chapman & Hall/CRC; 1994.

[20] Tonda T, Satoh K, Nakayama T, Katanoda K, Sobue T, Ohtaki M A nonparametric mixed-effects model for cancer mortality. Aust N Z J Stat. 2011;53:247–56. 10.1111/j.1467-842X.2011.00615.x

[21] Fotheringham AS, Brunsdon C, Charlton M. Geographically Weighted Regression: The Analysis of Spatially Varying Relationships. New York: Wiley; 2003.

[22] Satoh K, Yanagihara H Estimation of varying coefficients for a growth curve model. Am J Math Management Sci. 2010;30:243–56. 10.1080/01966324.2010.10737787

[23] Nakaya T, Fotheringham AS, Brunsdon C, Charlton M. Geographically weighted Poisson regression for disease association mapping. Stat Med. 2005;24:2695–717. 10.1002/sim.2129 16118814

[24] Nakaya T, Fotheringham AS, Charlton M, Brunsdon C. Semiparametric geographically weighted generalised linear modelling in GWR4.0. Proceedings of Geocomputation, 2009; online: 1–5.

[25] R Core Team. R: A language and environment for statistical computing. Vienna, Austria: R Foundation for Statistical Computing; 2014. Available from: http://www.R-project.org/.

[26] Satoh K, Yanagihara H, Kamo K Statistical inference on a linear varying coefficient on longitudinal data of discrete distribution. Jpn J Appl Stat. 2009;38:1–11 (in Japanese) 10.5023/jappstat.38.19

[27] Tonda T, Satoh K, Yanagihara H Statistical inference on a varying coefficient surface using interaction model for spatial data. Jpn J Appl Stat. 2010;39:59–70 (in Japanese) 10.5023/jappstat.39.59

[28] Tonda T, Satoh K, Otani K, Sato Y, Maruyama H, Kawakami H, . Investigation on circular asymmetry of geographical distribution in cancer mortality of Hiroshima atomic bomb survivors based on risk maps: analysis of spatial survival data. Radiat Environ Biophys. 2012;51:133–41. 10.1007/s00411-012-0402-4 22302183 PMC3332363

[29] Satoh K, Tonda T Estimating semiparametric varying coefficients for geographical data in a mixed effects model. J Japan Stat Soc. 2014;44(1):25–41. 10.14490/jjss.44.25

[30] Marugame T, Mizuno S. Mortality trend of lung cancer in Japan 1960–2000. Jpn J Clin Oncol. 2003;33:148–9. 12710458

[31] Ito Y, Ioka A, Nakayama T, Tsukuma H, Nakamura T. Comparison of trends in cancer incidence and mortality in Osaka, Japan, using an age-period-cohort model. Asian Pac J Cancer Prev. 2011;12(4):879–88. 21790220

[32] Kamo K, Kaneko S, Satoh K, Yanagihara H, Mizuno S, Sobue T. A mathematical estimation of true cancer incidence using data from population-based cancer registries. Jpn J Clin Oncol. 2007;37(2):150–5. 10.1093/jjco/hyl143 17272318

[33] Akita T, Ohisa M, Kimura Y, Fujimoto M, Miyakawa Y, Tanaka J. Validation and limitation of age–period–cohort model in simulating mortality due to hepatocellular carcinoma from 1940 to 2010 in Japan. Hepatol Res. 2014;44(7):713–9. 10.1111/hepr.12177 23730747

[34] Marugame T, Kamo K, Sobue T, Akiba S, Mizuno S, Satoh H, . Trends in smoking by birth cohorts born between 1900 and 1977 in Japan. Prev Med. 2006;42:120–7. 10.1016/j.ypmed.2005.09.009 16271753

[35] Takahashi H, Okada M, Kano K. Age-period-cohort analysis of lung cancer mortality in Japan, 1960–1995. J Epidemiol. 2001;11:151–9. 10.2188/jea.11.151 11512571 PMC11735078

[36] Tango T Estimation of age, period and cohort effects: decomposition into linear trend and curvature components. Jpn J Appl Stat. 1985;14:45–9 (in Japanese) 10.5023/jappstat.14.45

