# Supplementary material for: Detecting a Local Cohort Effect for Cancer Mortality Data Using a Varying Coefficient Model
Source: J Epidemiol. 2015 Nov 5;25(10):639–46. doi: 10.2188/jea.JE20140218 (PMC4626393; doi:10.2188/jea.JE20140218)
Supplement: Abstract in Japanese. [file je-25-639-s001.pdf]

変化係数モデルを用いたがん死亡データに対する局所的コホート効果の検出

冨田哲治[1]、佐藤健一[2]、加茂憲一[3]

[1] 県立広島大学 経営情報学部
[2] 広島大学 原爆放射線医科学研究所
[3] 札幌医科大学 医療人育成センター

【背景】がん死亡リスクは加齢とともに増大傾向にあり、高齢化が進む日本において、がん死亡リスクの評価や予測は重要な課題である。がん死亡リスクに影響を及ぼす時間要因は 3 つあり、その 1 つが出生コホート効果である。これまで、記述疫学研究により日本のがん死亡リスクにおけるコホート効果の存在が報告されている。
【方法】本研究では、出生コホート効果を自動検出し、その効果を統計的に評価するための方法を開発する。
【結果】提案法を日本人男性の肝臓がんと肺がんに関する死亡データに適用した。その結果、
肝臓がんについては 1934 年前後の出生コホートに有意な正の効果が認められ、その相対危険度は最大で約 1.54 倍であった。肺がんについては、1939 年前後の出生コホートに有意な負の効果が認められ、その相対危険度は最小で約 0.83 倍であった。
【結論】提案法で検出された出生コホート効果は、記述疫学研究の結果とほぼ一致しており、提案法の有用性が確認された。提案法の適用により、これまで見落とされている可能性がある弱い出生コホート効果の検出が期待される。

キーワード：出生コホート効果、日本のがん死亡、変化係数モデル
